# Bridging metabolic reprogramming and targeted therapy: the critical role of S-palmitoylation in cancer

**DOI:** 10.3389/fcell.2026.1802109

**Published:** 2026-03-19

**Authors:** Yan Sun, Weiqiang Tang, Ye Xia, Min Xia, Gaohua Liu, Wenjie Zhang, Yajun Chen, Jing Zhong

**Affiliations:** 1 Institute of Clinical Medicine, The First Affiliated Hospital, Hengyang Medical School, University of South China, Hengyang, Hunan, China; 2 Multi-Omics Research Center for Brain Disorders, Department of Neurology, The First Affiliated Hospital, Hengyang Medical School, University of South China, Hengyang, Hunan, China; 3 Department of Metabolism and Endocrinology, The Second Affiliated Hospital, Hengyang Medical School, University of South China, Hengyang, Hunan, China; 4 Cancer Research Institute, The First Affiliated Hospital, Hengyang Medical School, University of South China, Hengyang, Hunan, China

**Keywords:** epigenetic regulation, immune evasion, metabolic reprogramming, S-palmitoylation, targeted therapy, tumor microenvironment

## Abstract

Metabolic reprogramming provides cancer cells with excess fatty acids (FA) to adapt to metabolic stress; however, the precise mechanisms by which these lipid substrates are converted into sustained oncogenic signaling outputs remain incompletely elucidated. This article highlights S-palmitoylation, a reversible post-translational modification (PTM), as a critical molecular bridge linking substrate supply to protein membrane anchoring, stability, and activity. Notably, this interaction forms a malignant positive feedback loop: metabolic reprogramming expands the substrate pool, while aberrant S-palmitoylation conversely stabilizes metabolic enzymes, further exacerbating metabolic disruption. Mechanistically, dysregulated S-palmitoylation not only directly sustains key signaling pathways (RAS/MAPK, PI3K/AKT, and Hippo pathways) to promote stress tolerance but also regulates epigenetic plasticity, synergistically driving tumorigenesis, metastasis, and drug resistance. Beyond intracellular signaling, S-palmitoylation reshapes the tumor microenvironment (TME) by regulating the transport and degradation of immunomodulatory factors, notably promoting immune evasion by inhibiting the lysosomal degradation of programmed death-ligand 1 (PD-L1). This review synthesizes recent advances through three unique organizing pillars: (i) the bidirectional metabolic-palmitoylation feedback loops, (ii) palmitoylation-driven epigenetic plasticity, and (iii) the paradigm shift toward substrate-centric therapeutic designs, aiming to overcome current clinical challenges and enhance the efficacy of immunotherapy.

## 1 Introduction

Tumorigenesis is a multifaceted process driven by genetic mutations, epigenetic alterations, and the dysregulation of signaling pathways that govern cancer cell proliferation, survival, and metastasis (Zhao Y. et al., 2022). Despite the wide application of therapies targeting kinases or receptors, drug resistance and immune evasion remain significant clinical challenges. A hallmark of this adaptive resistance is metabolic reprogramming, particularly dysregulated lipid metabolism (Hanahan, 2022). To support rapid tumor growth, cancer cells exploit the Warburg effect and upregulate *de novo* lipogenesis (DNL), resulting in the accumulation of substantial pools of acetyl-CoA and fatty acids (FA) (Pavlova et al., 2022). However, these excess metabolic intermediates are not merely energy sources; they serve as critical signaling precursors that directly regulate protein stability and oncogenic signaling (Kim et al., 2019; Liu Y. et al., 2022).

**FIGURE 1:**
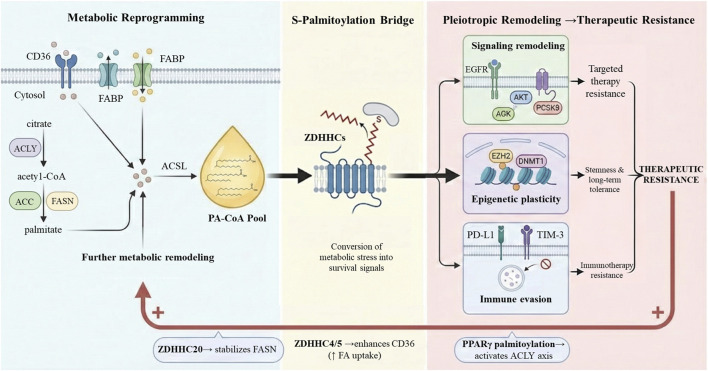
S-palmitoylation bridges bidirectional loops between metabolic reprogramming and therapeutic resistance. Exogenous FA uptake and DNL expand the intracellular PA-CoA pool. ZDHHC enzymes utilize this metabolic substrate to execute S-palmitoylation, driving pleiotropic remodeling across signaling, epigenetic, and immune axes, ultimately leading to targeted and immunotherapy resistance. Concurrently, a positive feedback loop is established: specific palmitoylation events (e.g., *via* ZDHHC20/4/5 and PPARγ) reciprocally amplify FA uptake and DNL, sustaining a highly malignant and drug-tolerant phenotype. Supported by BioRender (https://app.biorender.com).

S-palmitoylation emerges as the pivotal molecular bridge linking this metabolic state to cellular signaling. Unlike other lipid modifications, S-palmitoylation is unique for its reversibility, involving the covalent attachment of palmitate to cysteine residues via labile thioester linkages (Jin et al., 2021; Fan et al., 2024). This dynamic turnover is orchestrated by “Writers,” the zinc finger DHHC-type containing (ZDHHC) protein family and “Erasers,” the palmitoyl-protein thioesterases (PPTs), including acyl-protein thioesterases (APTs), and α/β hydrolase domain–containing proteins (ABHDs), enabling the cell to sensitively monitor intracellular palmitoyl-CoA (PA-CoA) levels (Rana et al., 2018). Consequently, S-palmitoylation acts as a central bridge converting lipid-induced “metabolic stress” into “survival signals,” establishing a bidirectional loop between metabolic reprogramming and therapeutic resistance (Figure 1). Specifically, the expanded PA-CoA pool fuels ZDHHC-mediated remodeling of signaling, epigenetic, and immune networks, while specific palmitoylation events reciprocally amplify lipid metabolism (Rocks et al., 2010). Such dynamic properties, along with tight control over palmitoylation states, enable rapid cellular adaptation to environmental changes and contribute to the maintenance of homeostatic equilibrium (Liu Z. et al., 2022).

**FIGURE 2:**
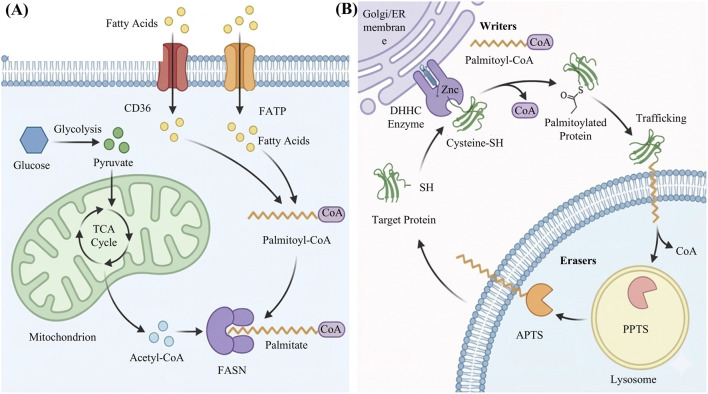
Metabolic basis and enzymatic mechanism of the S-palmitoylation cycle. **(A)** Dynamic cycling mechanism. S-palmitoylation links lipid metabolism to protein localization via reversible thioester bonds. ZDHHC palmitoyl acyltransferases mediate the transfer of palmitate from PA-CoA to substrate proteins at the endoplasmic reticulum and Golgi apparatus. Conversely, APTs and PPTs hydrolyze these bonds at cellular membranes and in lysosomes, enabling dynamic turnover. **(B)** Metabolic substrate supply. Glycolysis and the tricarboxylic acid cycle generate acetyl-CoA, which fuels *de novo* lipogenesis catalyzed by FASN. In parallel, CD36 and Fatty Acid Transport Proteins mediate exogenous fatty acid uptake, while ACS convert free palmitate into the donor substrate, PA-CoA. Supported by BioRender (https://app.biorender.com).

Accumulating evidence indicates that aberrant S-palmitoylation plays a critical role in the tumor microenvironment (TME) (Zhou et al., 2022). On one hand, dysregulated ZDHHC enzymes hijack the expanded FA pool to stabilize critical oncogenes (e.g., RAS, PI3K/AKT, and Hippo), thereby amplifying oncogenic signaling (Aomatsu et al., 2008; Liu Y. et al., 2022) (Figure 2A). On the other hand, S-palmitoylation reconfigures the immune landscape by inhibiting the ubiquitin-mediated degradation of immune checkpoints, such as programmed death-ligand 1 (PD-L1), facilitating immune escape and T cell exhaustion (Lu et al., 2019). Furthermore, S-palmitoylation mediates a feedback loop between metabolic reprogramming and epigenetic plasticity by regulating subcellular localization and the activity of epigenetic enzymes, thereby driving therapeutic resistance, metastasis, and relapse (Yao et al., 2019; Zhou et al., 2022).

Recent reviews have primarily summarized the basic enzymatic structures of ZDHHCs and their isolated signaling cascades. This review explicitly expands beyond these foundational aspects. S-palmitoylation integrates metabolic status, signal transduction, and immune evasion. Targeting this modification cycle offers a viable strategy to overcome current therapeutic bottlenecks. We systematically summarize the multidimensional regulatory roles of S-palmitoylation in cancer. We organize the literature around three conceptual pillars. These pillars include the bidirectional metabolic-palmitoylation feedback loops, palmitoylation-driven epigenetic plasticity, and the substrate-centric paradigm shift in therapeutic design. This framework explicitly integrates the impact of S-palmitoylation on tumor immune escape. It also highlights emerging substrate-targeted applications, including PROTACs, offering new insights into overcoming drug resistance and potentiating immunotherapy efficacy.

## 2 Metabolic control of S-palmitoylation in the TME

The distribution of proteins in cells is largely subject to S-palmitoylation and its cyclic state. The cyclic state is not completed by a certain enzyme alone, but is maintained by the ZDHHCs family and depalmitoylases together (Li et al., 2025). The continuous operation of the enzymatic reaction directly depends on the supply level of lipid substrates. In order to meet the demand for this raw material, tumor cells use metabolic reprogramming to actively reshape the internal fatty acid bank (Ali et al., 2018). The TME pressure generated by hypoxia or lack of nutrition will also further regulate the substrate supply system through metabolic stress networks (Chen et al., 2024). Thus, enzymatic activity and fatty substrate abundance together serve as the twin pillars that oversee the overall levels of S-palmitoylation.

### 2.1 Reciprocal regulation of lipid metabolism and S-palmitoylation

At the level of lipid metabolism, tumor cells markedly activate the DNL pathway in a process that involves citrate transfer from the mitochondria into the cytoplasm for cleavage into acetyl-CoA, which is then converted into endogenous palmitic acid (PA) via the coordinated action of ATP–citrate lyase (ACLY), acetyl-CoA carboxylase (ACC), and fatty acid synthase (FASN). However, free PA cannot be directly used for modification. It must be activated into biologically active PA-CoA by acyl-CoA synthetase (ACS). Clinical studies have shown that ACLY, ACC, and FASN are highly expressed across a range of solid and hematologic malignancies, and global upregulation of this lipogenic machinery is closely associated with poor prognosis (Yang et al., 2019; Nelson et al., 2020; Wan et al., 2025). Mechanistically, this aberrantly activated synthetic axis not only supplies a continuous stream of lipid building blocks for tumor growth but also markedly expands the PA-CoA substrate pool. At the downstream level, this enlarged pool enhances S-palmitoylation of key signaling proteins and further drives invasive behavior and malignant progression (Simeone et al., 2021; Zhou et al., 2023).

Tumor cells do not rely solely on endogenous synthesis, however, and this point will be further elaborated in the following sections. They also build an exogenous route for lipid supply by upregulating membrane transporters that capture FA from the TME. For instance, the transmembrane receptor CD36 and several fatty acid-binding proteins (FABPs) are markedly overexpressed in breast, ovarian, and other cancers, which enables efficient uptake of extracellular FA. FABP4 is a striking example. As a lipid chaperone, it helps ovarian cancer cells harvest FA directly from neighboring adipocytes and uses them to support tumor growth (Wan et al., 2025). CD36 overexpression appears particularly important in breast cancer, where it not only increases fatty acid uptake but also correlates with distant metastasis and poor prognosis (Yang et al., 2019) Regardless of whether FA comes from DNL or from exogenous sources, they must be converted into PA-CoA by members of the ACS family, such as ACSL1–6, before they can enter the S-palmitoylation cycle (Chen et al., 2025) (Figure 2B). In colorectal cancer and glioblastoma, abnormal expression of ACSL4 and ACSL5 has been reported to enhance fatty acid activation and, by altering the intracellular ACS landscape, to directly influence the efficiency of downstream protein palmitoylation (Cheng et al., 2016).

S-palmitoylation does not have a simple inverse relationship with lipid metabolism. S-palmitoylation even finely adjusts the function and stability of metabolic enzymes of crucial importance, thereby setting up a closed positive feedback loop. ZDHHC20-mediated S-palmitoylation of FASN has been illustrated to halt the ubiquitin-proteasome destruction of its highly lipogenic capacity in hepatocellular cancer (Mo et al., 2024). At the level of fatty acid uptake, palmitoylation of extracellular CD36 by ZDHHC4 and ZDHHC5 also plays a crucial role by stabilizing the accumulation of CD36 on the plasma membrane and significantly increasing its affinity for the uptake of exogenously introduced FA. Furthermore, palmitoylation of the transcription factor PPARγ activates the ACLY-related signaling pathway in activating lipid synthetic pathways, further boosting lipid production in colorectal cancer cells (Shan et al., 2024). Thus, S-palmitoylation and lipid metabolism do not exist in a simple unidirectional supply relationship, but rather in a tight positive feedback loop. Metabolic reprogramming not only constantly supplies the substrate needed for S-palmitoylation but also stabilizes and enhances the function of metabolic enzymes of crucial importance. This, in turn, ultimately promotes highly productive lipid metabolism in a way that allows the two processes to reinforce each other and act synergistically in driving tumor progression.

### 2.2 Dynamic S-palmitoylation under microenvironmental stress

The pathophysiological characteristics of the TME include tissue hypoxia, impaired nutrient transport and oxidative stress. These factors work together to form a complex metabolic stress environment, which in turn affects the steady-state maintenance of S-palmitoylation across multiple pathways (Jiang et al., 2018). In addition, these stress factors can not only alter the availability of lipid substrates but also directly modulate catalytic function of modifying and depalmitoylating enzymes.

Another central feature of the TME is hypoxia. At one level, hypoxia induces transcription of hypoxia-inducible factor 1α (HIF-1α), which activates sterol regulatory element-binding protein-1 (SREBP-1), thereby inducing expression of many of the lipid synthesis enzymes, including FASN (Lee et al., 2017). With regards to the regulation of S-palmitoylation, this metabolic pathway plays an active role in increasing the supply of PA-CoA within a cell. The increase in intracellular PA-CoA supply forms the required substrate basis for substrate-dependent protein modification. Several studies have demonstrated that, in the context of human glioblastoma, a metabolite of the transforming growth factor-β (TGF-β) pathway named palmitoylated SMAD family member-3 (Smad3) can engage in functional synergy with the HIF-1α signaling axis, both of which facilitate the shift of cancer cells to a mesenchymal state (Bollu et al., 2015). On the other hand, hypoxia can interfere with this modification as well, through pathways that do not in any way rely on HIF-1α. Indeed, one of the major hypoxic stressors leads to intracellular accumulation of nitric oxide (NO), which induces S-nitrosylation of H-RAS. This modifies H-RAS by allosteric means, leading to depalmitoylation and subsequent detachment from the cell membrane, thus abrogating its signaling function. In essence, the same stress cue can facilitate or abolish palmitoylation depending on the molecular target at play.

Nutritional deficiency can also induce cancer cells to activate their own palmitoylation compensation system. For example, glutamine deficiency can lead to an increase in the level of palmitylation of malate dehydrogenase 2 (MDH2) mediated by ZDHHC18, especially in ovarian cancer (Li et al., 2023). This change enhances the enzyme activity of lactate dehydrogenase 2 (LDH2), thus maintaining mitochondrial respiratory activity and giving cancer cells the ability to survive and adapt to nutritional deficiency conditions. In addition, under the condition of cell unfolded protein reaction (UPR), ZDHC9-mediated BiP/GRP78 palmitylation can inhibit the excessive activation of endoplasmic reticulum stress signals, thus helping bladder tumor cells maintain protein homeosis (Li et al., 2024).

Changes in reactive oxygen species (ROS) levels exert a direct bidirectional regulatory effect on palmitoylation homeostasis. High ROS can oxidize the cysteine residue in the catalytic center of ZDHHC enzymes, reduce palmitoyl acyltransferase activity, and at the same time enhance depalmitoylase activity. The net result is a global decrease in S-palmitoylation efficiency (Li et al., 2023). Yet certain S-palmitoylation events are integrated into the antioxidant defense system. S-palmitoylation of aquaporin 4 (AQP4), for example, lowers the permeability of the plasma membrane to hydrogen peroxide (H_2_O_2_) and thereby feeds back to limit intracellular oxidative stress (Cao et al., 2023).

Beyond local microenvironmental cues, systemic physiological rhythms of the host can remodel the S-palmitoylation network at the whole-body level. Disruption of circadian rhythm is closely associated with cancer progression (Lee, 2021). Mechanistic work has shown that sleep deprivation activates ACSL1 in a CLOCK-dependent manner and elevates systemic PA-CoA levels. The excess substrate then promotes S-palmitoylation of the CLOCK protein itself in a ZDHHC5-dependent way, creating a positive feedback loop that accelerates lung tumorigenesis (Peng et al., 2024). This finding shows, in a very direct way, that chronic disturbance of physiological rhythms can reshape the S-palmitoylation network by jointly altering the substrate pool and the activity of key enzymes.

## 3 Regulation of key oncogenic signaling pathways by S-palmitoylation

**FIGURE 3:**
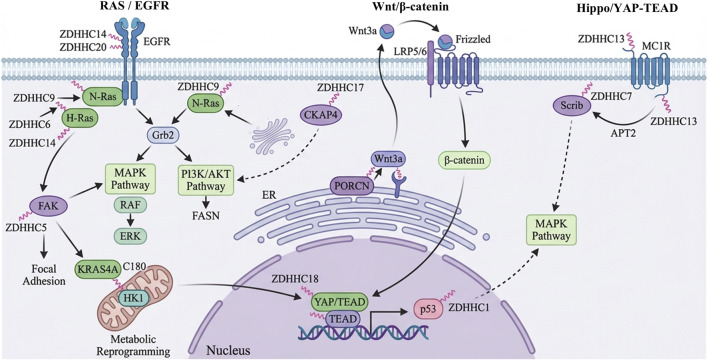
S-palmitoylation-driven oncogenic signaling networks and pathway crosstalk play a critical role in tumorigenesis. Aberrant S-palmitoylation functions as a central node in tumorigenesis by modulating protein dynamics. It facilitates RAS membrane anchoring to activate the MAPK cascade and modifies EGFR to stimulate the PI3K/AKT/mTOR pathway. Concurrently, S-palmitoylation precisely regulates the activity and subcellular localization of key components within the Wnt/β-catenin, Hippo/YAP–TEAD, and TGF-β signaling pathways. Collectively, these interactions establish a coordinated oncogenic network that sustains malignant cellular phenotypes. Supported by BioRender (https://app.biorender.com).

S-palmitoylation provides substrate proteins with a hydrophobic interface and, in doing so, builds a precise spatial framework for intracellular signal transduction. The membrane recruitment and complex assembly of oncogenic kinases such as RAS and PI3K/AKT depend directly on the dynamic balance of this lipid modification (Kadry et al., 2021). Canonical cascades that govern cell fate and tissue homeostasis, including the Wnt and Hippo pathways, are also constrained by the S-palmitoylation status of their core effectors (Li Q. et al., 2020). Pathways such as TGF-β, JAK/STAT and p53 use S-palmitoylation to rapidly adjust cell phenotype and stress responses (Liu Z. et al., 2022). In this section, we focus on how S-palmitoylation, through a shared mechanism of membrane recruitment, allosteric activation and dynamic cycling, exerts systematic control over pathway activity, drug resistance, and crosstalk between signaling networks (Figure 3). This lipid modification maintains critical signal transduction homeostasis in normal physiological states. Tumor cells actively hijack these identical modification cycles to drive malignant progression. A systematic comparison reveals the stark contrast between the physiological functions and oncogenic consequences of S-palmitoylation across these core signaling networks (Table 1).

**TABLE 1:** S-palmitoylation-mediated regulation of key signaling pathways: Physiological homeostasis *versus* oncogenic dysregulation.

Signaling pathway	Key substrates	Regulating enzymes	Physiological role (normal cells)	Oncogenic consequence (tumor cells)
RAS/MAPK	N-RAS, H-RAS, K-RAS4A, EGFR	ZDHHC9, 6, 14, 20/APT1, APT2, ABHD17	Regulates essential membrane-to-Golgi shuttling; controls amplitude and duration of normal growth signals	Drives hyperactivated plasma membrane anchoring; confers intrinsic resistance to EGFR TKIs and KRAS inhibitors
PI3K/AKT/mTOR	AKT, PCSK9, mTOR	ZDHHC17, 24, 16, 22	Mediates controlled, PIP3-dependent kinase activation and normal anabolic metabolism	Enables PIP3-independent AKT membrane anchoring; drives PTEN degradation; fosters Sorafenib and Tamoxifen resistance
Wnt/β-catenin	LRP6, Wnt ligands, CKAP4	ZDHHC19, 2/PORCN/APT1	Controls required ER-to-plasma membrane transport of receptors; regulates normal Wnt ligand secretion	Induces chronic LRP6 membrane localization; maintains EMT; promotes PORCN-inhibitor bypass mechanisms
Hippo/YAP-TEAD	Scrib, TEAD	ZDHHC7/APT2	Restricts YAP in cytoplasm via Scrib membrane-anchoring; strictly controls organ size homeostasis	APT2-mediated Scrib delocalization activates YAP; TEAD auto-palmitoylation stabilizes oncogenic YAP-TEAD transcription
JAK/STAT	STAT3	ZDHHC7/APT2	Enables cyclic membrane-to-nucleus shuttling for transient, controlled inflammatory responses	Promotes constitutive STAT3 activation and chronic inflammatory tumor microenvironment remodeling
TGF-β, GPCR & Other Signals	p53, Fas, MC1R	ZDHHC1, 7, 13/APT1, APT2	Directs p53 nuclear import; enables Fas-clustering for apoptosis	Drives TGF-β-mediated invasion; retains p53 in cytosol; evades apoptosis

This table outlines the specific substrates and regulating enzymes across core signaling networks, explicitly contrasting their essential functions in maintaining normal cellular homeostasis with their dysregulated roles in driving malignant progression.

### 3.1 Regulation of the RAS/MAPK signaling pathway

The RAS family mainly comprises three closely related isoforms—H-RAS, N-RAS and K-RAS—and their oncogenic mutations are well established as key drivers of human malignancies, with K-RAS being the most frequently mutated isoform in tumors. RAS signals primarily through the MAPK cascade, a central module that regulates cell growth, differentiation and survival. The oncogenic activity of RAS depends on a precise membrane cycling process mediated by S-palmitoylation (Busquets-Hernández and Triola, 2021). The tight coupling between membrane residency and signaling output also makes the palmitoylation cycle a plausible intervention point in RAS-dependent settings.

ZDHHC9 has been identified as a palmitoyl acyltransferase (ZDHHC) that mediates S-palmitoylation of N-RAS at Cys181 and H-RAS at Cys181/Cys184 (Liu et al., 2016). Monopalmitoylation of N-RAS at Cys181 drives vesicular transport from the endoplasmic reticulum to the plasma membrane, whereas dual palmitoylation of H-RAS at Cys181 and Cys184 fine tunes its distribution within membrane microdomains (Parker and Mattos, 2015). This regulatory network, however, shows marked enzymatic redundancy and compensation. Deletion of ZDHHC9 markedly weakens RAS driven transformation but does not fully abolish N-RAS palmitoylation, because related enzymes such as ZDHHC6 and ZDHHC14 can be upregulated and maintain a basal level of modification that is sufficient for tumor cell survival (Liu et al., 2016). In parallel, depalmitoylating enzymes including APT1/2 and members of the ABHD17 family remove the lipid moiety in a dynamic way and mediate RAS shuttling between the Golgi apparatus and the plasma membrane, thereby controlling both the amplitude and duration of downstream signaling (Busquets-Hernández and Triola, 2021). Such compensation implies that the impact of inhibiting a single ZDHHC isoform will differ across tumor contexts and may be limited in settings with robust backup activity. The KRAS4A isoform exhibits unique substrate specificity. Palmitoylation at Cys180 defines its mitochondrial localization and facilitates interaction with hexokinase 1 (HK1) and acts, through metabolic reprogramming, in tumor formation. Still, the specific palmitoyltransferase (PAT) responsible for this modification remains to be identified (Amendola et al., 2019).

This regulation extends beyond downstream effectors to upstream receptors, significantly impacting the Epidermal Growth Factor Receptor (EGFR). The palmitoylation of EGFR directly impacts the conformational stability of the receptor and its sensitivity to drugs. The mechanism involved in this control is a two-pronged one: We have previously demonstrated that FASN—a component of the signaling pathways involved in lung cancer—promotes palmitoylated EGFR at Cys797, which improves the stability and activity of the receptor (Ali et al., 2018). Conversely, ZDHHC20 specifically modifies the disordered C-terminal region (Cys1025/Cys1122) of EGFR. This modification induces steric hindrance that impedes Grb2 adaptor protein binding and inhibits E3 ubiquitin ligase recruitment, ultimately reducing receptor degradation and conferring tumor cell resistance to EGFR tyrosine kinase inhibitors (TKIs) (Runkle et al., 2016). Even when EGFR is wild type, S-palmitoylation can promote receptor dimerization and keep prosurvival signaling running (Thomas et al., 2019). Once EGFR is activated at the membrane, palmitoylation also helps further down the line by stabilizing the oncoprotein CDCP1, shielding it from lysosomal degradation and favoring its recycling back to the cell surface, which then feeds back into stronger oncogenic signaling (Adams et al., 2015). These multifaceted effects underscore that S-palmitoylation is not merely a passive anchor but a dynamic driver of TKI resistance, suggesting that profiling the EGFR palmitoylation state could serve as a vital predictive biomarker for patient stratification in targeted therapies.

Seen from this angle, it is not surprising that the S-palmitoylation network itself is emerging as a druggable node in RAS/EGFR-driven cancers. In K-RAS-mutant lung cancer, blocking ZDHHC20 lowers EGFR palmitoylation, selectively dampens the PI3K/AKT pathway and reduces MYC levels, and in this way can restore sensitivity to PI3K inhibition (Kharbanda et al., 2020). Resistance to K-RAS inhibitors frequently involves bypass activation of NRAS/HRAS or EGFR, and these bypass routes are highly dependent on S-palmitoylation. Combination strategies that co-target RAS signaling and palmitoylation are therefore attracting attention. For example, the ZDHHC20 inhibitor SD-066–4 shows robust antitumor activity in K-RAS-mutant models (Lee et al., 2024), while the FASN inhibitor orlistat reduces PA-CoA production at the source and enhances the response to TKI (Ali et al., 2018). Together, these findings suggest that disrupting specific interactions between substrate pools and ZDHHC enzymes is a key step toward reversing compensatory resistance in RAS/EGFR-driven cancers.

### 3.2 Regulation of the PI3K/AKT/mTOR signaling pathway

The PI3K/AKT/mTOR axis is a central hub that controls cell proliferation, metabolism and survival. Under classical activation, growth factors such as EGF and IGF stimulate receptor tyrosine kinases, leading to activation of phosphatidylinositol 3 kinase (PI3K). PI3K generates PIP3, which recruits 3 phosphoinositide dependent protein kinase 1 (PDK1) and AKT to the plasma membrane, where AKT becomes phosphorylated and subsequently activates downstream targets including mTOR to support growth and anabolic metabolism (Peng et al., 2022).

However, AKT activation is anything but a canonical pathway. Accumulating evidence demonstrates that S-palmitoylation contributes greatly to the stability of AKT at the plasma membrane. The efficiency of AKT membrane anchoring, and thus how long the active state persists, no longer depends solely on the presence of PIP3 but has a direct relationship with its S-palmitoylation status (Blaustein et al., 2021). Elevated palmitate availability can strengthen AKT association with ZDHHC17 and ZDHHC24 and link AKT modification to the cellular palmitate pool. These enzymes have been reported to palmitoylate AKT at Cys77 and Cys224, which supports membrane localization and reduces inactive aggregation, thereby extending pathway output (Bu et al., 2024). This PIP3-independent membrane recruitment exposes a major clinical limitation of canonical PI3K inhibitors; tumors with upregulated lipid biosynthesis can bypass targeted PI3K blockade by directly anchoring AKT to the membrane, suggesting that dual inhibition of both PI3K and lipid metabolism is essential for durable efficacy. In hepatocellular carcinoma models, the FASN inhibitor orlistat reduces palmitate supply and has been used to suppress palmitoylation-associated AKT activation and tumor growth (Ali et al., 2018; Bu et al., 2024). This illustrates that selective strategies based on substrate deprivation, enzyme modulation, and kinase blockade are an acceptable treatment strategy for metabolically driven hepatocellular carcinoma.

Besides direct alteration of AKT, S-palmitoylation also establishes a complicated bidirectional regulatory network in the PI3K/AKT pathway at the level of the key proteins, proprotein convertase subtilisin/kexin type 9 (PCSK9) and mTOR. In sorafenib-resistant hepatocellular carcinoma cells, ZDHHC16 facilitated the palmitoyl modification of PCSK9 at the Cys600 site. The modification enhances the binding affinity between PCSK9 and the cancer-inhibiting protein PTEN, thus inducing the degradation of PTEN through the lysosome pathway. The deletion of PTEN lifts the inhibition of the PI3K signaling pathway, resulting in an increase in the level of AKT phosphorylation, and finally making tumor cells resistant to drugs (Sun et al., 2022). However, in breast cancer, S-palmitoylation has an anti-tumor effect. Specifically, ZDHHC22 specifically modifies the Cys361/362 site of mTOR, which destroys the stability of the mTOR complex, thus inhibiting the activation of downstream AKT. Another study reveals that the reactivation of the expression of ZDHHC22 significantly increases the drug susceptibilities of drug-resistant cells to tamoxifen (Huang et al., 2022). The specific modification differently mediated by various enzymes reflects the extremely plastic regulation competency of S-palmitoylation in the same signaling pathway. Besides, S-palmitoylation also mediates the cancerous cross-regulation between EGFR and PI3K pathways. In non-small cell lung cancer, the palmitoylation state of EGFR directly influences the intensity of its interaction with the PI3K regulatory subunit p85. The modification of EGFR by ZDHHC20 enhances the recruitment of heterogeneous PI3K dimers to the target membranes to amplify the downstream AKT signaling and ultimately fuel cancer cell proliferation (Kharbanda et al., 2020). In summary, S-palmitoylation shows remarkable plasticity in the regulation of kinase signaling pathways. It not only directly activates kinases such as AKT via conformational modulations but also indirectly affects their signal output by changing upstream regulators or key components of signaling pathways. Its ultimate impacts largely rely on the specific enzyme-substrate interaction and the innate properties of the tumor itself.

### 3.3 Regulation of the Wnt/β-catenin signaling pathway

The canonical Wnt/β-catenin signaling pathway is triggered by the binding of extracellular Wnt ligands to membrane-associated Frizzled receptors and co-receptors LRP5/6, leading to a cascade of intracellular signals that drives the accumulation of intracellular β-catenin, translocation of β-catenin into the nucleus, and transcriptional expression of proliferative genes (Xiang et al., 2025). S-palmitoylation impairs signaling in this pathway by strictly governing the subcellular localization and functional integrity of key signaling proteins. As a critical co-receptor to initiate the pathway, the localization of LRP6 in the membrane is directly regulated by S-palmitoylation at Cys1394 and Cys1399 and is required for transport of LRP6 from the endoplasmic reticulum to the plasma membrane, the absence of which causes receptor retention within cells that inhibits Wnt signaling (Abrami et al., 2008). In osteosarcoma cells, elevated ZDHHC19 levels retain LRP6 membrane localization and Wnt signaling chronically active, whereas loss of ZDHHC19 abolishes this anchoring and Wnt/β-catenin signaling (Liang et al., 2022).

S-palmitoylation can also function on the inhibitory side of the pathway through Wnt antagonists. For instance, Dkk1 enhances APT1-mediated depalmitoylation of the cytoskeletal protein CKAP4, resulting in the displacement of CKAP4 from the plasma membrane. Once CKAP4 is removed from the membrane, Wnt signaling is further inhibited, and CKAP4 is unable to effectively activate the PI3K/AKT pathway. ZDHHC2 restores palmitate to CKAP4, re-transporting it to the membrane. PI3K/AKT activity was stimulated again, although the degree was weak, and the anti-receptor function of Dkk1 was only partially restored (Planey et al., 2009; Sada et al., 2019; Harada et al., 2020). The role of acyl-CoA synthase 5 (ACSL5) in regulating the Wnt signaling pathway varies depending on the tissue type, while ACSL5 plays the opposite role in tumors. ACSL5 specifically converts the S-palmitoylation of Wnt2B, isolates it in the mitochondria, and finally prevents its transposition to the nucleus, thus reducing its Wnt signal activity and has a negative impact on cell proliferation. However, in acute myeloid leukemia, ACSL5 is palmylated and isolated Wnt3a, which directly stimulates the Wnt/β-catenin pathway and promotes malignant transformation (Klaus et al., 2014). Further research shows that this functional duality is not absolute, but depends on the availability of specific Wnt liand lines and metabolic substrates in the TME. The difference in lipid metabolism between the intestinal epithelium and the bone marrow microenvironment forces ACSL5 to selectively combine with different substrates, thus switching the functional state between tumor inhibition and tumor activation. This environment-dependent duality also exists within the ZDHHC enzyme family. Most ZDHHC isoforms drive oncogenesis. ZDHHC22, conversely, exhibits potent tumor-suppressive activity in breast cancer by destabilizing the mTOR complex and inhibiting downstream AKT signaling (Huang et al., 2022). These functional contradictions severely compromise the clinical feasibility of pan-ZDHHC inhibition. Broad-spectrum enzymatic blockade risks neutralizing intrinsic tumor-suppressive mechanisms alongside oncogenic pathways. Such indiscriminate disruption creates unpredictable clinical responses and introduces significant systemic toxicities across normal tissues. Future clinical translation must shift away from global pan-suppression. Target development requires highly selective intervention strategies tailored to specific tumor contexts.

Membrane-bound O-acyltransferase Porcupine (PORCN) is responsible for S-palmitoylation of Wnt ligands and thus controls their maturation and secretion. PORCN inhibitors such as LGK974 and, ETC-159, which block this rate-limiting step, have already shown promising antitumor activity in clinical trials (Shah et al., 2021). Tumor cells, however, can acquire resistance through compensatory routes. In colorectal cancer, upregulation of Wnt2B in cooperation with Frizzled7 enables cells to bypass PORCN inhibition and maintain epithelial-mesenchymal transition (EMT) (Schwab et al., 2018). In prostate cancer, overexpression of FASN provides excess palmitate, increases S-palmitoylation of Wnt1, stabilizes β-catenin, and promotes tumor progression (Fiorentino et al., 2008).

### 3.4 Regulation of the Hippo/YAP–TEAD signaling pathway

The Hippo pathway controls organ size and tissue homeostasis through a kinase cascade that ultimately restrains the nuclear transcriptional activity of YAP/TAZ. S-palmitoylation acts as a spatial regulator in this system and defines the physical boundaries of signaling both at the plasma membrane and in the nucleus.

Upstream in the cascade, the tumor suppressor Scrib functions as a scaffold that keeps Hippo kinase complexes in the right place at the right time. When ZDHHC7 attaches a palmitate chain to Scrib, the protein is locked onto the plasma membrane, where it helps assemble LATS1/2 and favors phosphorylation and cytoplasmic retention of YAP; at the same time, this membrane-bound pool of Scrib tones down PI3K/AKT and MAPK signals (Johnson and Halder, 2014). After APT2 removes the lipid label, the Scrib protein is detached from the cell membrane and abnormally located in the cell. This abnormal spatial redistribution destroys the inhibitory activity of Hippo pathways and lifts the regulatory restrictions on carcinogenic pathways such as PI3K/MAPK, which eventually leads to the formation of malignant tumors (Hernandez et al., 2017). At the downstream level, the function of the transcription factor Transcriptional Enhanced Associate Domain (TEAD) depends on a fragile structure-activity coupling. The TEAD protein possesses a conserved hydrophobic pocket that is auto-S-palmitoylated to fill the pocket and induce a structural reorganization that forms the binding pocket for YAP/TAZ association (Noland et al., 2016). When this modification is missing, TEAD adopts a less stable shape, becomes a preferred target of the ubiquitin–proteasome system, and is cleared more rapidly, which makes it unable to efficiently recruit YAP and drive transcription of genes that support cell growth and survival (Mills et al., 2024).

This structural dependency has made the internal hydrophobic cavity of TEAD an attractive druggable site. Small-molecule inhibitors such as JM7 and K-975 occupy the palmitoylation pocket, block the binding site required for lipidation and in doing so prevent TEAD palmitoylation. This destabilizes TEAD, disrupts YAP–TEAD complex formation at its origin and has shown notable antitumor activity in several preclinical models (Li et al., 2022). At the same time, long-term suppression of a single node can introduce new problems. When TEAD function is chronically impaired, tumor cells may activate compensatory feedback circuits, for instance by upregulating Wnt/β-catenin signaling and using β-catenin to take over part of the YAP-dependent transcriptional program, thereby establishing alternative resistance routes. In parallel, cancer cells may adjust the expression of lipid metabolic enzymes and shift the substrate spectrum available for TEAD palmitoylation, allowing some residual transcriptional activity to persist. Thus, by controlling the membrane localization of Scrib and the structural stability of TEAD, S-palmitoylation regulates Hippo pathway output at both cytoplasmic and nuclear levels. This dual control underlines why the pathway is so sensitive to changes in palmitoylation dynamics.

### 3.5 Regulation of TGF-β, GPCR and other oncogenic signals

Beyond the major growth pathways discussed above, the S-palmitoylation network extends deeply into signaling routes that govern differentiation, cell death, and immune responses. In glioblastoma, ZDHHC19-mediated S-palmitoylation of Smad3 activates TGF-β signaling and induces expression of mesenchymal markers, which supports an invasive tumor phenotype (Fan et al., 2021). Hedgehog signaling also depends on specific lipid modifications. Hedgehog acyltransferase catalyzes S-palmitoylation of Sonic Hedgehog at Cys24. The small molecule inhibitor RU-ski 43 blocks this enzyme and effectively suppresses Hedgehog driven tumor growth (Resh, 2021).

Many membrane receptors and their downstream components rely on lipid anchoring for functional stability. In melanoma, the G protein-coupled receptor (GPCR) MC1R maintains tumor-suppressive activity through ZDHHC13-mediated S-palmitoylation at Cys315 (Wang et al., 2022). Cancer-associated MC1R variants such as R151C disrupt this modification and impair receptor function, whereas inhibition of the depalmitoylating enzyme APT2 can restore MC1R activity (Chen et al., 2019). The tumor suppressor role of the G protein subunit GNA13 is regulated in a similar fashion. In B cell lymphoma, S-palmitoylation of GNA13 at Cys14 and Cys18 is required for its plasma membrane localization and activation of downstream Rho signaling, which helps restrain malignant transformation (Xia et al., 2021).

Cell survival and inflammatory responses are also shaped by dynamic acylation cycles. Fas receptor-induced apoptosis depends on the formation of stable receptor clusters at the plasma membrane, a process driven by ZDHHC7-mediated S-palmitoylation at Cys199 (Rossin et al., 2015). Chronic lymphocytic leukemia cells overexpress APT1 and APT2, remove this modification, and thereby prevent Fas clustering and escape apoptosis (Berg et al., 2015). The JAK/STAT pathway uses the palmitoylation cycle to control the flow of signals between membrane and nucleus. ZDHHC7-mediated S-palmitoylation enhances membrane recruitment and phosphorylation of Signal Transducer and Activator of Transcription 3 (STAT3), while subsequent APT2-mediated depalmitoylation releases phosphorylated STAT3 from the membrane and allows its nuclear translocation. This cyclic process helps explain why STAT3 remains persistently active in many tumors (Zhang et al., 2020).

Subcellular trafficking of nuclear tumor suppressors is likewise under strict palmitoylation control. For p53, full transcriptional activity depends on S-palmitoylation at several cysteine residues, including Cys135, catalyzed by ZDHHC1. Without this modification, p53 fails to efficiently accumulate in the nucleus and cannot properly activate downstream targets such as p21 (Tang et al., 2021). In summary, these findings underscore that S-palmitoylation is not merely a marginal modification; it pervades almost all of the major cell-fate programs, from membrane receptors such as GPCRs to nuclear transcription factors like p53, and from death receptor signaling *via* Fas to inflammatory JAK/STAT pathways. A deeper understanding of the wiring of individual steps in palmitoylation in these networks will be required to design pathway- and tumor-type–appropriate interventions that target palmitoyl acyltransferases of interest or their substrates.

## 4 Regulation of tumor epigenetic plasticity by S-palmitoylation

The capacity of tumor cells to survive and adapt to a changing microenvironment is reliant on the ability to dynamically reprogram their gene expression programs. Such epigenetic flexibility is undoubtedly related to chromatin-modifying enzyme activity and distribution. However, an increasing body of evidence shows that S-palmitoylation appears to occupy a strategic position at the crossroads of signal transduction, metabolic regulation, and epigenetic regulation and seems to play a central role in connecting these three processes (Zhang et al., 2021). ZDHHC family members directly employ core epigenetic regulators such as histone methyltransferases as substrates and modulate their stability and nuclear localization by covalent modification. Conversely, several epigenetic factors modified by S-palmitoylation can regulate ZDHHC expression or enzymatic activity. This reciprocal regulation over time leads to the establishment of close regulatory circuits composed of positive and negative feedback loops that maintain the malignant phenotype, reconstitute the epigenomic landscape continuously and, ultimately, contribute to therapeutic resistance (Tang et al., 2024).

### 4.1 Indirect control through signaling pathways

The contribution of S-palmitoylation to epigenetic plasticity does not solely depend upon a direct action on epigenetic enzymes. A more likely scenario is that S-palmitoylation influences upstream signaling pathways first, and this change, in turn, rewrites the epigenetic state. Acting as a molecular switch regulating pivotal avenues, including PI3K/AKT and RAS/MAPK, S-palmitoylation markedly contributes to the activation status of most central kinases in these networks (Charollais and Van Der Goot, 2009). Once AKT or ERK is persistently activated, the signal progresses in a stepwise way to the nucleus. It has been observed that AKT or ERK, which are continuously activated, relay the signal by means of phosphorylation of key epigenetic enzymes to modify their catalytic activity, stability, and subcellular localization. This creates a cascade of regulation that stretches from membrane sensors and cytoplasmic kinases to chromatin modulators that constitute a common pathway in malignant transformation (Nussinov et al., 2025).

**FIGURE 4:**
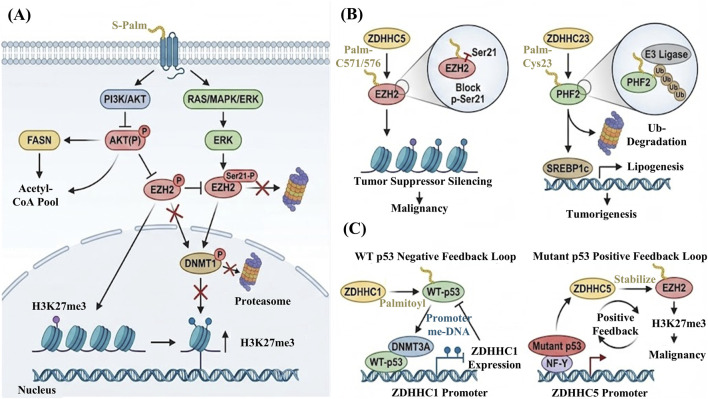
S-palmitoylation as a molecular bridge between metabolism and epigenetic regulation. **(A)** Indirect regulation. S-palmitoylation of membrane receptors triggers PI3K/AKT and MAPK signaling cascades, leading to the phosphorylation of epigenetic modifiers (e.g., EZH2 and DNMT1) and subsequent alterations in the chromatin state. **(B)** Direct modification. ZDHHC enzymes directly palmitoylate chromatin regulators such as EZH2 and PHF2, thereby modulating their protein stability and enzymatic activity. **(C)** Bidirectional feedback loops. The wild-type p53/ZDHHC1 axis forms a self-limiting negative feedback loop to maintain homeostasis, whereas the mutant p53/ZDHHC5 axis establishes a reinforcing positive feedback circuit that solidifies an aggressive epigenetic program. Supported by BioRender (https://app.biorender.com).

The example of the regulation of the enzyme histone methyltransferase EZH2 demonstrates such a mechanism. The phosphorylation signals from AKT and ERK pathways that are directed to Enhancer of zeste homolog (EZH2) are opposing and even functionally distinct. AKT activates Ser 21 of EZH2 for phosphorylation, which greatly diminishes the enzymatic activity of the EZH2 methyltransferase and in turn changes the pattern of distribution of the inhibitory mark H3K27me3 in the genome. ERK pathway activation induces another phosphorylation pattern that speeds up proteolytic degradation of EZH2 (Li Z. et al., 2020). Combined, they lead to the dynamic balance involving EZH2 abundance on chromatin paired with functional output. Persistent activation of AKT blocks proteasomal degradation of DNA methyltransferase 1 (DNMT1) through phosphorylation-dependent mechanisms and thereby stabilizes DNMT1 protein (Agarwal et al., 2013). In this setting, high DNMT1 expression, supported indirectly by S-palmitoylation, allows tumor cells to maintain long-term silencing of key tumor suppressor genes and drives tumor progression at the epigenetic level (Robert et al., 2003) (Figure 4A). Class II histone deacetylases (HDACs) are another group of direct substrates of AKT and MAPK. Their phosphorylation status controls nuclear–cytoplasmic shuttling and, as a result, determines whether they can enter the nucleus to enforce transcriptional repression (Yao et al., 2019).

Beyond signaling itself, S-palmitoylation also reshapes metabolic networks that provide essential cofactors for chromatin modification. Enzymes such as FASN are directly regulated by S-palmitoylation, which in turn affects intracellular acetyl-CoA production (Das et al., 2024). Because acetyl-CoA is the obligatory donor for histone acetylation, its abundance sets an upper limit on the degree of chromatin acetylation (Eduardo et al., 2025). As discussed in Section 3.4, changes in Hippo/YAP–TEAD signaling further influence the recruitment of histone acetyltransferases (HATs) to target loci.

In summary, S-palmitoylation establishes a critical indirect regulatory chain. It indirectly alters the phosphorylation states of EZH2, DNMTs, and HDACs by activating the AKT/MAPK signaling pathway. At the same time, it indirectly controls the substrate supply for histone acetylation through metabolic regulation. This dual indirect regulation of signaling switches and metabolic supply lays the foundation for the reprogramming of the epigenetic state.

### 4.2 Direct S-palmitoylation of epigenetic enzymes

Compared with indirect control, direct S-palmitoylation of epigenetic enzymes offers a faster and more specific way to adjust their behavior. By modifying these proteins at defined cysteine residues, S-palmitoylation can immediately influence their stability, localization, and catalytic activity, and in this way rewrite the epigenetic code with high precision.

ZDHHC5 and ZDHHC16 often act as positive regulators in this context. They enhance the function of target enzymes by blocking inhibitory modifications or preventing degradation. In p53 mutant glioma, ZDHHC5 specifically mediates S-palmitoylation of EZH2 at Cys571 and Cys576. The lipid anchor occupies nearby space and interferes with access to Ser21, thereby preventing inhibitory phosphorylation at this site. As a result, EZH2 remains highly active, global H3K27me3 levels rise, and glioma stem cells maintain strong self-renewal capacity and tumorigenic potential (Chen et al., 2017). A similar stabilizing effect has been reported for the histone methyltransferase SETD2. In glioblastoma with EGFR amplification, ZDHHC16-mediated S-palmitoylation protects SETD2 from ubiquitin-proteasome system-mediated degradation. This modification keeps SETD2 protein levels stable and allows efficient DNA damage responses that help preserve genome integrity (Fan X. et al., 2022).

In contrast to the stabilizing cases described above, ZDHHC23 exerts an opposing effect on a subset of epigenetic regulators. In hepatocellular carcinoma, the histone demethylase PHF2 is S-palmitoylated on Cys23, and this small chemical tag does not protect the protein at all; instead, it boosts its ubiquitination and accelerates proteasome-dependent turnover. As PHF2 levels fall, repression of the lipogenic transcription factor SREBP1c is lifted, leading to abnormal lipid accumulation and driving malignant proliferation (Jeong et al., 2023) (Figure 4B). It is worth noting that this degradation still exists even in microenvironments rich in PA, and only by inhibiting ZDHHC23 can the anti-cancer effect of PHF2 be restored. These examples emphasize that direct palmitylation is not one-way. For some enzymes, it stabilizes and activates its function by blocking the degradation pathway by spatially blocking. For other enzymes, it labels proteins and accelerates their degradation. The final result depends on the specific enzyme–substrate pair and the modified cysteine site. This diversity broadens the toolkit available for epigenetic regulation and highlights the central role of S-palmitoylation in shaping the tumor epigenomic landscape, while also offering a conceptual basis for isoform-selective ZDHHC inhibitors as targeted therapies.

### 4.3 Feedback loops between palmitoylation and epigenetic regulation

The relationship between palmitoylation enzymes and their substrates is not a one way chain of commands. Instead, many pairs form tightly coupled feedback loops. In normal cells, these loops help maintain signal balance; in tumor cells, they are often rewired to stabilize malignant traits.

In the setting of wild type p53, cells use a DNA methylation-based negative feedback circuit to limit pathway activity. ZDHHC1-mediated S-palmitoylation of p53 at Cys135, Cys176, and Cys275 is required for nuclear translocation of p53 and for activation of tumor suppressor genes (Tang et al., 2021). Once in the nucleus, p53 does more than execute a simple anticancer program. It also recruits DNMT3A to the promoter of the ZDHHC1 gene. DNMT3A induces hypermethylation of this promoter and silences ZDHHC1 transcription, which in turn lowers ZDHHC1 protein levels. Reduced ZDHHC1 expression diminishes p53 palmitoylation and nuclear import, and the p53 pathway is gradually shut down. Under physiological conditions, this negative feedback loop prevents excessive p53 activation. During early tumorigenesis, however, cancer cells exploit the same mechanism to evade p53 mediated immune surveillance and growth arrest (Le et al., 2020; Tang et al., 2021).

In glioblastoma, one striking example of this logic is a self-amplifying loop between ZDHHC4 and glycogen synthase kinase 3β (GSK3β). ZDHHC4 palmitoylates GSK3β on Cys14, and this seemingly small change reshapes its phosphorylation pattern: inhibitory Ser9 phosphorylation falls, while activating Tyr216 phosphorylation goes up. Activated GSK3β then engages the EZH2–STAT3 axis and drives binding of STAT3 to the ZDHHC4 promoter, increasing ZDHHC4 transcription (Kim et al., 2013). This positive feedback loop allows glioma cells to maintain high EZH2 activity and a stable stem-like phenotype over long periods.

Meanwhile, the genetic background of the mutation transforms the nature of the feedback loops. In p53-mutated gliomas, mutant p53 no longer functions as a tumor repressor; instead, it assembles with the transcription factor NF-Y to directly activate the transcription of ZDHHC5. The upregulation of ZDHHC5 enhances palmitoylation and stabilization of EZH2, thereby potentiating the H3K27me3-mediated gene silencing outcome (Chen et al., 2017) (Figure 4C). In the same molecular pair functioning in a feedback loop, contrasting genomic and microenvironmental configurations induce different shifts in the direction of the regulation as well as in the functional output, testifying to the strong dependency of tumor epigenetic plasticity on specific cellular environments and genetic backgrounds.

## 5 Functional impact of S-palmitoylation on malignant tumor phenotypes

In functional terms, S-palmitoylation equips tumor cells with a set of highly adaptive malignant traits. As a key link between lipid metabolism and protein function, this modification contributes to stemness maintenance, drug tolerance and acquisition of invasive and metastatic behavior. Inside the cell, S-palmitoylation stabilizes core factors such as FAK and Oct4A and strengthens survival and stemness related signaling (Zhou et al., 2023). At the cell surface, the same chemistry modifies immune checkpoints including PD-L1 and TIM-3, blunting antitumor immune responses (Yang et al., 2019; Zhang et al., 2024). The combined effect on intracellular oncogenic signaling and extracellular immune evasion machinery fundamentally increases the plasticity and resilience of tumor cells under therapeutic pressure.

### 5.1 Regulation of tumor cell plasticity

Tumor plasticity encompasses the transition of cells from a differentiated state to a dedifferentiated state, adaptation to therapeutic stress (resistance), and EMT. These seemingly independent malignant phenotypes are, in fact, synergistically driven by a network of S-palmitoylation modifications. This modification continuously activates core survival signals such as PI3K/AKT, establishing an integrated mechanism whereby a single modification drives multiple malignant phenotypes.

Aberrant activation of the PI3K/AKT pathway is a key node that links resistance and invasion. In hepatocellular carcinoma, ZDHHC16 catalyzes S-palmitoylation of the proprotein convertase PCSK9 at Cys600. This modification does not act in isolation; it increases the binding affinity between PCSK9 and the tumor suppressor PTEN and promotes PTEN trafficking to lysosomal degradation. The absence of PTEN lifts the inhibition of the PI3K/AKT signaling pathway, resulting in the continuous activation of AKT. Functionally, this transformation supports sorafenid resistance and may enhance cell migration and invasiveness through the downstream AKT effect (Sun et al., 2022). A similar trend was observed in clear cell renal cell carcinoma, in which ZDHHC2-activated acylglycerol kinase (AGK) undergoes palmitylation modification at the Cys72 site. palmitoylation modified AGK is more likely to approach the cell membrane, forming a signal complex and directly activating the PI3K/AKT/mTOR pathway, thus giving cancer cells tolerance to sunitinib and enhancing their survival (Sun et al., 2023). These findings support palmitoylation as a localization switch, but they also suggest that biomarker strategies may need to track subcellular distribution or modification state rather than total protein abundance alone. The activation of kinase driven by palmitoylation is another key mechanism for promoting EMT. In glioblastoma, ZDHHC5 specifically activates the palmityl modification of non-receptor tyrosine kinase FAK in Cys456 site. This makes FAK firmly fixed on the cell membrane, thus effectively activating and phosphorylizing the downstream PI3K/AKT signaling pathway. The activation of this signaling pathway not only promotes cell proliferation, but also directly activates EMT-related transcription procedures, giving cancer cells a stronger aggressive ability (Wang Y. et al., 2024). It is worth noting that changes in depalmylase activity are also involved in the formation of these processes. In cervical cancer, the balance between ZDHHC19 and PPT1 is broken, resulting in palmitoylation of Flotillin-1. This then stabilized the IGF-1 signaling pathway and upregulation of the migration factor TIAM1, which together promoted the EMT process. Specifically, the stabilization of IGF-1 signaling and upregulation of TIAM1 collaboratively promote EMT progression (Kwon et al., 2023).

Beyond migration and invasion, maintaining a pool of stem-like cells with self-renewal capacity is fundamental to long term tumor survival, relapse and distant metastasis. S-palmitoylation also plays a central role here by stabilizing key transcription factors and activating self-renewal pathways. In glioblastoma stem cells, ZDHHC4 mediated palmitoylation of GSK3β at Cys14 enhances self-renewal and stabilizes downstream signaling networks, which contributes to resistance to temozolomide (Zhao C. et al., 2022). ZDHHC17 directly modifies the stemness factor Oct4A. This modification protects Oct4A from lysosomal degradation. Researchers validated this Oct4A regulatory axis utilizing patient-derived glioblastoma stem cells and orthotopic mouse xenograft models (Chen et al., 2023). A growing body of preclinical studies have shown that S-palmitoylation is involved in the maintenance of stem cells in various solid tumors and has been functionally confirmed as a key dependent pathway in glioblastoma cells.

### 5.2 Regulation of the tumor immune microenvironment

Tumor cells maintain a high intracellular palmitate pool by activating fatty acid DNL and increasing uptake of exogenous FA. This metabolic adaptation provides a continuous supply of substrate for S-palmitoylation. Beyond meeting biosynthetic needs, it supports the modification of key immunoregulatory proteins and helps build a multilayered immunosuppressive network within the TME.

The first line of this network is control of immune checkpoint stability and resistance to degradation at the plasma membrane. For PD-L1, S-palmitoylation at Cys272 is a central mechanism that prevents lysosomal degradation. Although the dominant palmitoyl acyltransferase exhibits tumor-type specificity, with ZDHHC3 predominating in colorectal cancer and ZDHHC9 in breast and lung cancer, the core mechanism remains consistent: the steric hindrance imposed by the palmitoyl moiety impedes recognition by E3 ubiquitin ligases, thereby inhibiting ubiquitin-dependent endosomal sorting and degradation (Yao et al., 2019; Lin et al., 2023). In pancreatic cancer, high ZDHHC9 expression is closely linked to impaired antitumor immunity. Genetic deletion of ZDHHC9 can convert a “cold” TME into a “hot” one, slow tumor progression and extend survival. This effect depends on CD8^+^ T cells and sensitizes tumors to PD-L1 blockade (Lin et al., 2023). ZDHHC9 also modifies PD-1 on the tumor cell surface, maintaining its abundance and, independently of ligand interaction, activating mTOR signaling to promote tumor growth (Du et al., 2021; Yao et al., 2021). When people talk about T-cell exhaustion, they usually focus on PD-1, but S-palmitoylation quietly pushes this process forward by tuning other co-inhibitory receptors as well. For the checkpoint receptor TIM-3, palmitoylation at Cys296 blocks its interaction with the E3 ligase Hrd1, so the protein is not efficiently ubiquitylated and cleared. As a result, TIM-3 accumulates abnormally on T cells and NK cells (Zhang et al., 2024), and its persistent high expression accelerates functional exhaustion. Clinical observations show that TIM-3 palmitoylation in melanoma correlates positively with expression of exhaustion markers such as PD-1 and LAG3 (Sakuishi et al., 2010), pointing to a critical role for this modification in driving the TME toward immune tolerance. Overall, by restricting ubiquitin mediated degradation, S-palmitoylation stabilizes immune checkpoints at the membrane, raises the activation threshold for T cells and speeds their progression into exhaustion.

S-palmitoylation is also key on the pro-immune front of the signaling axis to ensure the integrity of interferon-γ (IFN-γ) signaling, as the subunit IFNGR1 requires S-palmitoylation at Cys122 to avert AP3D1-mediated sorting into the lysosome (Du et al., 2021). In the innate immune compartment, the cyclic cGAS–stimulator of interferon genes (STING) pathway is also part of this dynamic cycle. Palmitoylation of cGAS, promoted by ZDHHC9, facilitates its dimerization and DNA-sensing ability, thereby activating STING signaling. Depalmitoylating enzymes including APTs, PPTs, and in particular PPT1 then remove the modification and thus prevent excessive inflammation (Fan et al., 2023). In hepatocellular carcinoma and melanoma, when PPT1 is hyperactive, it silences cGAS signaling and directs the polarization of macrophages toward the M2 phenotype. Pharmacologic PPT1 inhibitors GNS561 and DC661 induce lysosomal lipid peroxidation and restore type I interferon secretion, thus reprogramming the TME toward a proinflammatory state (Xu et al., 2022; Bhardwaj et al., 2023). Thus, while S-palmitoylation stabilizes immune checkpoint proteins such as PD-L1 and TIM-3 on the one hand, it conversely disrupts signaling integrity at the IFNGR1/cGAS axis, provoking immune escape from a systemic viewpoint. From a therapeutic engineering standpoint, strategies targeting the inhibition of these depalmitoylating enzymes might not only disrupt direct survivability of the tumor cells but also reprogram the TME with increased sensitivity to immunotherapy, creating an excellent biological rationale for future combinatorial application with established immune checkpoint blockade.

## 6 Therapeutic strategies targeting S-palmitoylation

Given the central role of S-palmitoylation in shaping malignant phenotypes and promoting immune escape, targeting this dynamic modification cycle has become an attractive direction in cancer therapy. The field is gradually moving away from early broad-spectrum inhibitors toward more focused, substrate-centered strategies. The first wave of compounds was hampered by pronounced off-target toxicity and limited selectivity (Han et al., 2025), which made clinical translation difficult. More recently, new covalent inhibitors and PROTAC-based approaches have made it possible to shut down individual ZDHHC isoforms with much higher precision (Lu et al., 2023). In parallel, pharmacologic targeting of depalmitoylating“Collectively, enzymes such as PPT1 has shown the capacity to reshape lysosomal homeostasis and activate antitumor immunity (Li et al., 2023). Strategies that occupy substrate binding pockets or mimic palmitoylation motifs further help to circumvent resistance driven by enzymatic redundancy. This section summarizes the evolution from non-specific enzyme inhibition to substrate-precise interference (Table 2).

**TABLE 2:** Therapeutic targeting of palmitylation mechanisms in cancer.

Target	Strategy	Specific target	Mechanism of action	Clinical potential	Ongoing clinical trials/Status	References
ZDHHCs	2-BP	Broad-spectrum	Irreversible covalent binding to ZDHHC active site	Inhibits NRF2/β-catenin signaling; limited by off-target toxicity	Preclinical	Fan X. et al. (2022), Hu et al. (2022), Wang Q. et al. (2024)
	CMA	Broad-spectrum	Acrylamide derivative targeting cysteine thiols	Blocks CD36/EGFR signaling with reduced toxicity	Preclinical	Azizi et al. (2021), Azizi et al. (2022)
	Artemisinin	ZDHHC6	Covalently inhibits ER-localized ZDHHC6	Reduces NRAS palmitoylation and oncogenic signaling	Phase II/III	Qiu et al. (2022)
	Curcumin	ZDHHC3	Inhibits enzyme auto-palmitoylation	Blocks ITGβ4 modification and membrane localization	Preclinical	Coleman et al. (2015)
	PROTAC	ZDHHC5 ZDHHC20	Induces UPS-mediated enzyme degradation	Downregulates IFITM3 palmitoylation; overcomes redundancy	Preclinical	Bai et al. (2024)
	SD-066–4	ZDHHC20	Potent, selective inhibition of ZDHHC20	Overcomes resistance: Blocks palmitoylation-dependent KRAS bypass signaling in mutant models	Preclinical	Lee et al. (2024)
Depalmitoylases	ML349	APT2	Selectively inhibits APT2 activity	Restores Scrib membrane localization; suppresses MAPK signaling	Preclinical	Dekker et al. (2010), Hernandez et al. (2017)
	GNS561/DC661	PPT1 (Lysosomal)	Inhibits PPT1; disrupts lysosomal homeostasis	Induces immunogenic cell death; sensitizes anti-PD-1 therapy	Phase I/II	Harding et al. (2022), Sharma et al. (2022), Xu et al. (2022)
	ABD957	ABHD17	Selective covalent inhibition of ABHD17	Traps NRAS in endomembranes; synergistic with MEK inhibitors	Preclinical	Remsberg et al. (2021)
Substrates	VT104/TM2	TEAD	Covalently occupies palmitate-binding pocket (PBP)	Destabilizes TEAD; blocks YAP/TAZ-TEAD complex assembly	Preclinical	Fan M. et al. (2022), Hu et al. (2022)
	Peptide Mimetics	ZDHHC3/PCSK9	Mimics substrate modification motifs (e.g., PD-L1)	Competitively inhibits specific substrate palmitoylation	Preclinical	Shi et al. (2024b), Wang Y. et al. (2024)
	LGK974, ETC-159	PORCN	Inhibits Wnt ligand acylation and secretion	Blocks Wnt/β-catenin signaling in solid tumors	Phase I/II	Shah et al. (2021)
	TVB-2640	FASN	Inhibits de novo lipogenesis; restricts PA-CoA pool	Restores EGFR TKI sensitivity; overcomes metabolic resistance	Phase II/III	Ali et al. (2018)

This table summarizes pharmacological strategies targeting ZDHHCs, depalmitoylases, and specific substrates, detailing their mechanisms of action in blocking oncogenic signaling pathways and their potential clinical applications.

### 6.1 Targeting palmitoyl acyltransferases

ZDHHCs act as the “writers” of S-palmitoylation. Through their conserved DHHC zinc finger domain, they catalyze transfer of lipid groups and thereby determine the membrane anchoring and functional activity of key oncoproteins such as RAS and EGFR. Because of their driver role in tumorigenesis, ZDHHCs were among the earliest therapeutic targets explored in this field. Broad-spectrum inhibitors were the first tools used, but their impact *in vivo* has been limited by a lack of specificity. The lipid analog 2-bromopalmitate (2-BP) remains the most widely used research compound. It forms an irreversible covalent bond with the catalytic cysteine of ZDHHC enzymes and blocks formation of the acyl-intermediate (Ko and Dixon, 2018). In preclinical models, 2-BP can suppress tumor growth driven by NRF2 and β-catenin, which strongly supports the idea that global S-palmitoylation is pro-tumorigenic. However, 2-BP reacts with many non-target proteins, including APT1, APT2, ACSs and glycerol-3-phosphate acyltransferases, and its cytotoxicity has prevented clinical translation (Davda et al., 2013; Abdulrahman et al., 2025).

To improve pharmacologic properties, non-lipid acrylamide derivatives such as CMA were developed. CMA uses a reactive warhead that preferentially engages cysteine thiols in the catalytic site and avoids the highly reactive ACS-derived intermediates associated with 2-BP. In this way, it reduces toxicity while still effectively blocking signaling through CD36 and EGFR (Azizi et al., 2022). These broad inhibitors have helped to confirm the antitumor potential of suppressing S-palmitoylation on a global scale, but their off-target effects highlight the need for isoform-selective agents. Importantly, ZDHHC enzymes also execute physiological functions in non-malignant tissues, which may further constrain systemic inhibition strategies. For example, ZDHHC2, ZDHHC7, and ZDHHC21 contribute to key steps in T cell activation and immune signaling, suggesting that sustained inhibition of selected isoforms could carry a risk of immunosuppression (Fan et al., 2020; Zhang et al., 2020; Lin, 2021). 2-BP directly perturbs mitochondrial membrane potential in non-malignant cells. This cellular toxicity clearly validates these safety concerns (Szkudelski and Szkudelska, 2011). At the same time, genetic deletion of specific ZDHHC isoforms has been reported to produce limited developmental phenotypes in some murine models (Marciel et al., 2018), which may indicate context-dependent dispensability and a potential therapeutic window. Collectively, these findings support a shift toward isoform-selective, substrate-informed, or tumor-specific delivery strategies, rather than prolonged pan-inhibition of the ZDHHC family. One practical example is EGFR-mutant NSCLC, where an EGFR TKI could be paired with FASN inhibition or ZDHHC20 targeting to limit palmitoylation-supported persistence and delay resistance.

Some natural or repurposed compounds have started to show better selectivity than classical broad-spectrum inhibitors. The antimalarial drug artemisinin (ART) can covalently inhibit endoplasmic reticulum–localized ZDHHC6, selectively reducing NRAS palmitoylation and blunting its oncogenic output (Qiu et al., 2022). Curcumin has been reported to interfere with the autopalmitoylation of ZDHHC3 and to block modification and membrane localization of integrin β4 (ITGβ4) (Coleman et al., 2015). These findings suggest that drug repurposing and natural product screening may help to overcome selectivity bottlenecks for ZDHHC inhibitors. To move beyond the inherent limits of small-molecule active site inhibitors, peptide-based and degradation-based strategies are now gaining momentum. Competitive peptide approaches mimic the palmitoylation motif of specific substrates and selectively disrupt enzyme–substrate interactions. For example, a cell-penetrating peptide carrying the PD-L1 palmitoylation sequence can compete with endogenous PD-L1 for binding to ZDHHC3, lower PD-L1 palmitoylation and promote its lysosomal degradation without significantly affecting other substrates (Shi et al., 2024a). Even more promising is the use of PROTAC technology to induce targeted degradation of ZDHHC enzymes. In engineered cell systems, Halo-PROTAC constructs have successfully driven ubiquitination and degradation of DHHC5 and DHHC20, leading to a marked reduction in palmitoylation of their transmembrane substrate IFITM3 (Bai et al., 2024). This “de-enzyme” strategy bypasses the problem of highly conserved active sites and provides a clean way to shut down specific ZDHHC-driven signaling programs in cancer.

### 6.2 Targeting depalmitoylating enzymes

The dynamic reversibility of S-palmitoylation is maintained by the coordinated activity of three families of “erasers”: APTs, PPTs and ABHDs. These depalmitoylating enzymes do more than simply remove lipid groups; they control the trafficking of signaling proteins such as RAS and Scrib between the plasma membrane and the endomembrane system. Because ZDHHC inhibitors often struggle with selectivity, serine hydrolase-type depalmitoylating enzymes, which have clearer structural features, have become attractive alternative targets. For cytosolic APTs, progress has been made toward isoform-selective inhibition and pathway-specific modulation. The early broad inhibitor Palmostatin B can interfere with the RAS depalmitoylation cycle but does not distinguish between APT isoforms. In contrast, APT family members are structurally amenable to isoform-selective blockade, which makes it possible to modulate certain substrates without globally disturbing the S-palmitoylation network. For example, ML349 selectively inhibits APT2-mediated depalmitoylation of Scrib, restores proper membrane localization of this tumor suppressor, and attenuates MAPK-driven malignant progression (Dekker et al., 2010; Hernandez et al., 2017). Isoform-selective agents of this kind not only help limit off-target toxicity but also offer a precise experimental handle to dissect substrate-specific regulatory events.

Lysosomal PPT1 inhibitors have revealed a distinct mode of action that couples organelle stress with immune activation. High PPT1 expression correlates with poor prognosis in hepatocellular carcinoma and oral squamous cell carcinoma. New small molecules such as GNS561 and DC661 do not primarily block upstream signal transduction. Instead, by inhibiting PPT1 they cause palmitoylated proteins to accumulate within lysosomes, disrupt lysosomal acidification and autophagic flux, and ultimately induce immunogenic cell death (Harding et al., 2022; Xu et al., 2022). Importantly, PPT1 inhibition also activates the cGAS-STING pathway in macrophages, increases type I interferon production and converts a “cold” TME into a “hot” one, thereby enhancing the response to anti-PD-1 therapy (Sharma et al., 2022). In this sense, PPT1 inhibitors act both as direct cytotoxic drugs and as potent immune sensitizers.

Within the ABHD family, covalent inhibitors of specific members have opened new options for combination therapy in hard-to-treat tumors. ABD957 is a highly potent inhibitor of the ABHD17 family and can selectively block NRAS depalmitoylation. As a result, NRAS becomes trapped in the endomembrane system and cannot recycle back to the plasma membrane for re-palmitoylation, which cuts off its oncogenic signaling at the source. Studies have shown that ABD957 synergizes with MEK inhibitors to produce a strong synthetic lethality effect in NRAS mutant acute myeloid leukemia (Remsberg et al., 2021). This supports ABHD17 inhibition plus MEK inhibition as a rational combination regimen in NRAS-driven disease, and it may also help when KRAS blockade selects for RAS-pathway bypass.

Depalmitoylating enzymes execute indispensable physiological roles in non-malignant tissues. APTs universally regulate basic cellular trafficking across healthy organs. PPT1 strictly governs lysosomal protein degradation within the central nervous system. PPT1 deficiency causes the abnormal accumulation of palmitoylated proteins and directly induces cellular apoptosis (Zhang et al., 2006; Zhao et al., 2019). Prolonged pharmacological PPT1 inhibition during cancer therapy poses a significant risk of cumulative neurotoxicity. This also supports tumor-specific combinations in ‘cold’ tumors, such as PPT1 inhibition or ZDHHC9 targeting with PD-1/PD-L1 blockade when PD-L1/PD-1/TIM-3 are high. ABHD17 enzymes dynamically control critical synaptic components like microtubule-associated protein 6 in primary neurons. This specific regulation maintains axonal stability and normal synaptic plasticity (Tortosa et al., 2017). Systemic blockade of these depalmitoylating enzymes threatens to disrupt these fundamental physiological cycles. Future clinical target development must prioritize tumor-targeted delivery systems or highly specific allosteric modulators. These advanced strategies will maximize anti-tumor efficacy and actively preserve essential neurological homeostasis.

### 6.3 Emerging substrate-targeted strategies

Given the substantial substrate-recognition overlap and enzymatic redundancy within the ZDHHC family, a rational alternative is to directly target defined palmitoylation sites or lipid-binding pockets on substrate proteins. Conceptually, this approach relies on using small molecules to occupy a substrate’s lipid-binding pocket or employing peptides that mimic the palmitoylation motif to competitively disrupt enzyme–substrate interactions, thereby selectively silencing oncogenic signaling without perturbing global S-palmitoylation levels.

Allosteric inhibition of the transcription factor TEAD is a prominent example. Drug development for the Hippo pathway has been hampered by the lack of clear binding pockets on YAP and TAZ, whereas TEAD contains a conserved palmitate-binding pocket (PBP) that is structurally well defined. New small molecules such as VT104, MYF-03-69 and TM2 do not inhibit upstream enzymes; instead, they covalently occupy the hydrophobic PBP within TEAD. This binding destabilizes TEAD folding, prevents its autopalmitoylation and promotes subsequent ubiquitin-proteasome system–mediated degradation. In doing so, these compounds disrupt assembly of the YAP/TAZ–TEAD transcriptional complex and shut down expression of its oncogenic target genes at the transcriptional source (Fan M. et al., 2022; Hu et al., 2022). This mechanism makes deliberate use of the structural dependence of TEAD on S-palmitoylation and achieves efficient, pathway-specific inhibition of Hippo signaling.

Competitive peptide strategies offer a complementary way to fine-tune membrane protein function. For the immune checkpoint PD-L1, researchers have designed a cell-penetrating peptide that contains the sequence surrounding its Cys272 modification site. This peptide competes with endogenous PD-L1 for binding to the catalytic pocket of ZDHHC3, reduces PD-L1 palmitoylation, and promotes its lysosomal degradation, thereby restoring T cell antitumor activity (Wang Q. et al., 2024). A similar concept has been applied to interfere with PCSK9 palmitoylation and, in doing so, to curb aberrant activation of the AKT pathway (Sun et al., 2022). Although peptide therapeutics still face practical challenges in terms of membrane permeability and *in vivo* half-life, their intrinsic high specificity offers a valuable route to target palmitoylation-dependent interfaces that are difficult to drug with conventional small molecules.

## 7 Discussion and therapeutic perspectives

Cancer remains a systemic disease driven by the complex interplay between metabolic adaptation and oncogenic signaling. As highlighted in this review, S-palmitoylation acts as a pivotal molecular hub that links metabolic substrate supply to protein functionality. By converting the “metabolic stress” of excess lipids into “survival signals,” this modification orchestrates a pro-tumorigenic network involving the membrane stabilization of RAS/EGFR/Hippo and the remodeling of the TME. While preclinical studies suggest that targeting this dynamic cycle offers a potent blockade against tumor growth, the transition from bench to bedside remains fraught with challenges.

A major hurdle for clinical translation lies in the structural complexity and functional redundancy of the ZDHHC family. The “one-to-many” and “many-to-one” relationships between enzymes and substrates create a robust compensatory network. For instance, inhibition of a single enzyme (e.g., ZDHHC9) often triggers compensatory activation of others (e.g., ZDHHC6 and ZDHHC14), leading to drug resistance (Quiroz et al., 2025). Moreover, due to the high conservation of catalytic cores, progress in developing isoform-selective inhibitors without off-target toxicity has been slow, and no single-target inhibitors have yet entered clinical trials (Remsberg et al., 2021). In addition to drug research and development, the lack of reliable biomarkers also limits the stratification of patients. At present, there is no antibody that can selectively identify site-specific S-palmitoylation epitopes. Relying on indirect biochemical exchange analysis (e.g., acyl-biotin exchange) not only complicates the experimental process, but also hinders the *in situ* evaluation of the modification level in clinical samples (Garst et al., 2021; Ocasio et al., 2024). Breaking through these detection bottlenecks is a prerequisite for establishing S-palmitoylation as a reliable prognostic biomarker. Crucially, it must be emphasized that S-palmitoylation does not have a universal tumor-promoting effect. Its functional consequences depend on the specific situation. Although it can anchor cancer genes such as RAS, it is also crucial to the function of cancer suppressor genes such as Scrib and p53. Therefore, broad-spectrum inhibition may inadvertently destroy the inherent inhibition mechanism of the tumor, resulting in inconsistent treatment response. Severe systemic toxicities necessitate this transition from global pan-suppression to tumor-specific interventions. Because both ZDHHC ‘writers’ and depalmitoylating ‘erasers’ also sustain essential homeostatic programs in non-malignant tissues, prolonged systemic inhibition can narrow the therapeutic window and complicate dose optimization.

Future research needs to overcome these clinical hurdles. The following specific questions will guide this translational process: How can researchers define tumor-type-specific palmitoylation landscapes in models that reflect patient disease and treatment exposure? Quantitative, site-resolved proteomics may help delineate enzyme–substrate networks and identify context-specific dependencies. How can researchers develop site-specific readouts that are suitable for biomarker translation? Chemical probes and targeted mass spectrometry could enable direct measurement of selected modification sites in clinical specimens, but their robustness and clinical utility require validation. How can clinicians design combination regimens that account for enzymatic redundancy and signaling compensation? Mechanism-guided combinations with EGFR- or MEK-directed agents, or with immune checkpoint blockade, should be tested in settings where palmitoylation contributes to pathway persistence or immune evasion. How can the field define a therapeutic window while preserving essential palmitoylation events in non-malignant tissues? Studies that map normal-tissue liabilities and compare them with tumor dependencies will be critical for selecting targets and dosing strategies. Emerging chemical proteomics tools directly address the limitations of site-resolved S-palmitoylation detection. Clickable palmitate analogs and acyl exchange-based enrichment techniques combined with mass spectrometry significantly improve coverage and site confidence (Vandekeere et al., 2026). Palmitoyltransferase chemogenetic systems enable the construction of precise enzyme-substrate pairings. ZDHHC-specific reporter molecules further visualize these dynamic modifications. These precise tools largely alleviate redundancy in ZDHHC family enzymes (Ocasio et al., 2024). Quantitative analyses can now assess specific palmitoylation sites in tumor biopsies and extracellular vesicles. These analyses provide crucial pharmacodynamic readouts in targeted therapy (Kong et al., 2023). They offer a viable technological pathway for tumor marker development and pharmacodynamic monitoring, but clinical validation remains in its early stages.

In summary, the dynamic cycle of S-palmitoylation serves as a crucial bridge between tumor metabolic reprogramming and malignant phenotypes. By overcoming current limitations in specificity and detection, and by integrating precise palmitoylation-targeting agents into existing chemo-immunotherapy regimens, we can pave the way for overcoming treatment resistance and significantly improving patient prognosis.

## Glossary

2-BP: 2-bromopalmitate
ABHDs: α/β hydrolase domain–containing proteins
ACC: Acetyl-CoA carboxylase
ACLY: ATP–citrate lyase
ACS: Acyl-CoA synthetase
AGK: Acylglycerol kinase
APTs: Acyl-protein thioesterases
DNL: *De novo* lipogenesis
TKI: Tyrosine kinase inhibitor
EGFR: Epidermal growth factor receptor
EMT: Epithelial-mesenchymal transition
EZH2: enhancer of zeste homolog
FA: Fatty acids
FABPs: Fatty acid binding proteins
FASN: Fatty acid synthase
GSK3β: Glycogen Synthase Kinase 3β
HIF-1α: Hypoxia-Inducible Factor 1α
MDH2: Malate dehydrogenase 2
PA: Palmitic acid
PA-CoA: Palmitoyl-CoA
PCSK9: Proprotein convertase subtilisin/kexin type 9
PD-L1: Programmed death-ligand 1
PORCN: Membrane-bound O-acyltransferase porcupine
PPTs: Palmitoyl-protein thioesterases
PTM: Post-translational modification
ROS: Reactive oxygen species
Smad3: SMAD family member-3
STAT3: Signal transducer and activator of transcription-3
STING: Stimulator of interferon genes
TEAD: Transcriptional enhanced associate domain
TGF-β: Transforming growth factor-β
TME: Tumor microenvironment
ZDHHCs: Zinc finger DHHC-type containing
